# Supplementary material for: Comprehensive Co-Inhibitory Receptor (Co-IR) Expression on T Cells and Soluble Proteins in Rheumatoid Arthritis
Source: Cells. 2024 Feb 26;13(5):403. doi: 10.3390/cells13050403 (PMC10931001; doi:10.3390/cells13050403)
Supplement: Supplementary file 1 [file cells-13-00403-s001.zip › cells-2832360-supplementary.pdf]

Comprehensive co-inhibitory receptors (Co-IRs) expression T cells and soluble proteins predict RA pathogenesis and activity

Chin-Man Wang*[1], Yeong-Jian Jan Wu*[2], Li-Yu Huang[2], Jian-Wen Zheng[2], Ji-Yih Chen[2]

Supplementary Figure S1: Representative gating strategy of CD4+CD127+/TIGIT+, CD8+CD127+/TIGIT+; CD8+HLA-DR+/CD38+; CD8+HLA-DR+CD127+, CD8+HLA-DR+CD38+

Supplementary Table S1: The clinical characteristics of RA patients & normal controls

Supplementary Table S2: The correlation between RA Disease Activity and one Co-IRs expression on T cells

Supplementary Table S3: The correlation between RA Disease Activity and Two Co-IRs expression on T cells

Supplementary Figure S1:

CD4+CD127+/TIGIT+, CD8+CD127+/TIGIT+

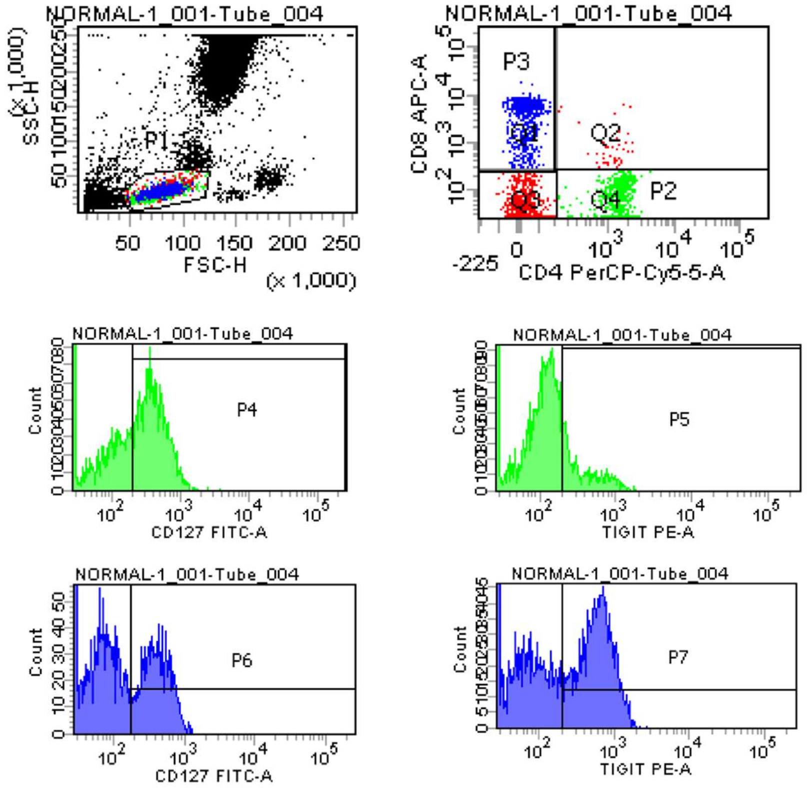

CD8+HLA-DR+/CD38+

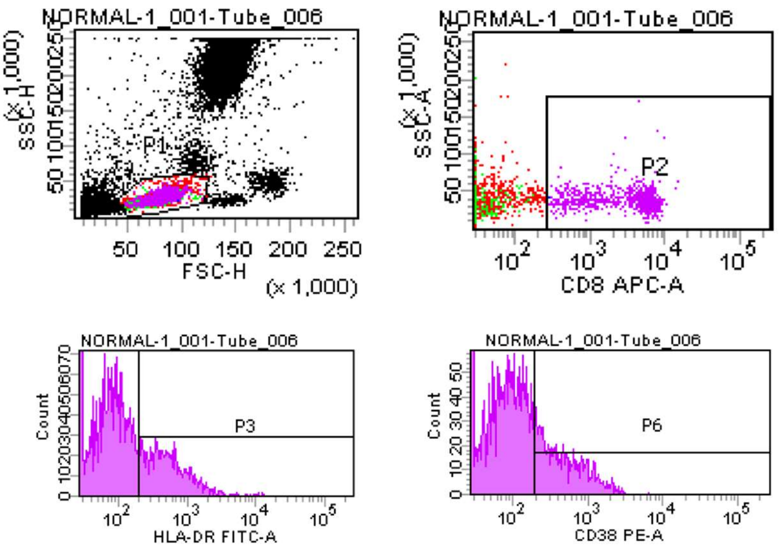

CD4+CD127+TIGIT+, CD8+CD127+TIGIT+

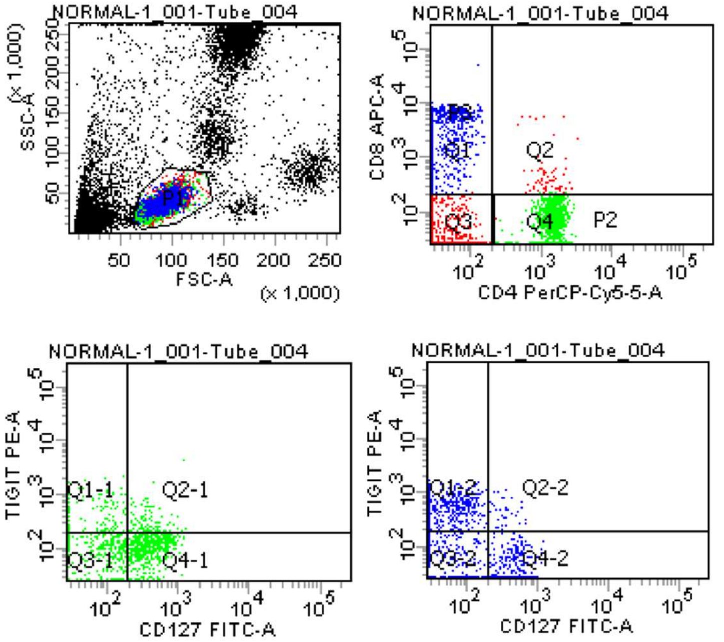

CD8+HLA-DR+CD127+, CD8+HLA-DR+CD38+

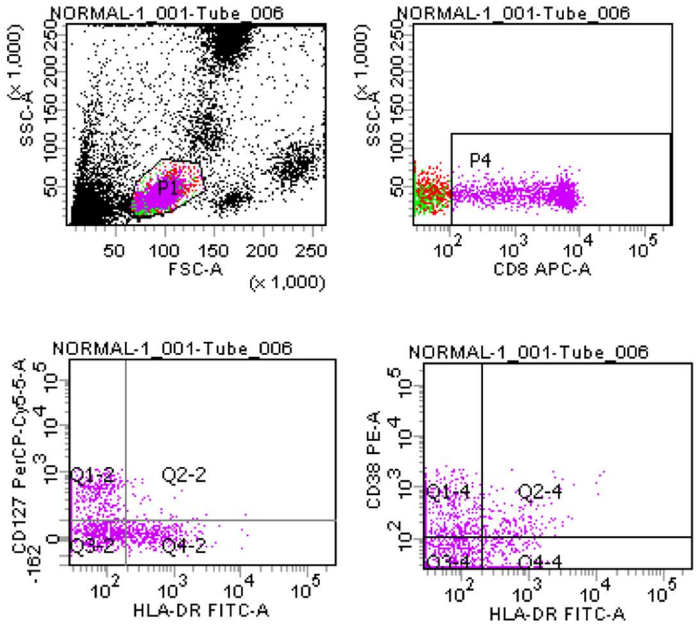

Supplementary Table S1: The clinical characteristics of RA patients & normal controls

Clinical parameters	RA (N=48)	Normal (N=27)
Age (year)	58.4±13.5	36.3±5.7
Male/female	10/38	8/19
RF (Positive >15/Negative <15)	32/16	
RF (>100/<100)	21/27	
ESR (mm/h)	43.3±31.8	
HS-CRP (mg/L)	24.3±26.0	
DAS28-ESR	5.4±1.7	
DAS28-CRP	4.9±1.6	
TCD3	72.1±10.3	
BCD19	9.0±4.7	
CD4/CD8 ratio	2.3±1.3	
RA X-ray stages		
I	11	
II	7	
III	14	
IV	16	
Methotrexate	28	
Sulfasalazine	37	
Hhydroxychloroquine	38	
Leflunomide	10	
Low dose steroid (<10mg)	36	
TNFα blocker	5	
JAK inhibitor	8	
Actemra	1	

Rheumatoid factor (RF) positive (>15 IU/mL) and high titers (>100 IU/mL)

Supplementary Table S2: The correlation between RA Disease Activity and one Co-IRs expression on T cells

T cells Co-IRs	ESR	HS-CRP	Das28 ESR	Das28 CRP
CD4+CD279+, %	r=0.2243, p=0.1136, N=51	r=0.2992, p=0.0251, N=56	r=0.2083, p=0.1647, N=46	r=0.2288, p=0.1219, N=47
CD4+CD279+, MFI	r=0.3594, p=0.0096, N=51	r=0.2762, p=0.0393, N=56	r=0.2323, p=0.1203, N=46	r=0.2261, p=0.1264, N=47
CD4+TIM3+, %	r=0.1537, p=0.2817, N=51	r=0.2679, p=0.0459, N=56	r=0.1237, p=0.4129, N=46	r=0.2144, p=0.1479, N=47
CD4+TIM3+, MFI	r=-0.1167, p=0.4148, N=51	r=-0.1202, p=0.3774, N=56	r=-0.2679, p=0.0719, N=46	r=-0.2792, p=0.0573, N=47
CD4+CTLA4+, %	r=0.1091, p=0.446, N=51	r=0.2021, p=0.1353, N=56	r=0.007428, p=0.9609, N=46	r=-0.00288, p=0.9847, N=47
CD4+CTLA4+, MFI	r=0.004822, p=0.9771, N=38	r=0.00154, p=0.9923, N=42	r=-0.1665, p=0.3317, N=36	r=-0.1144, p=0.5063, N=36
CD4+LAG3+, %	r=0.2133, p=0.1328, N=51	r=0.2299, p=0.0883, N=56	r=0.145, p=0.3365, N=46	r=0.1247, p=0.4036, N=47
CD4+LAG3+, MFI	r=0.02048, p=0.9002, N=40	r=-0.006626, p=0.966, N=44	r=-0.06727, p=0.6882, N=38	r=-0.0226, p=0.8929, N=38
CD4+CD127+, %	r=-0.1262, p=0.3774, N=51	r=-0.04402, p=0.7473, N=56	r=-0.2444, p=0.1016, N=46	r=-0.2642, p=0.0727, N=47
CD4+CD127+, MFI	r=-0.2014, p=0.2009, N=42	r=-0.1147, p=0.448, N=46	r=-0.3395, p=0.0321, N=40	r=-0.3839, p=0.0145, N=40
CD4+TIGIT+, %	r=0.05521, p=0.7004, N=51	r=0.138, p=0.3104, N=56	r=0.2487, p=0.0956, N=46	r=0.2341, p=0.1133, N=47
CD4+TIGIT+, MFI	r=0.2159, p=0.1753, N=41	r=0.3585, p=0.0156, N=45	r=0.156, p=0.3429, N=39	r=0.06448, p=0.6965, N=39
CD8+CD279+, %	r=0.2295, p=0.1052, N=51	r=0.2054, p=0.1288, N=56	r=0.2962, p=0.0456, N=46	r=0.3062, p=0.0363, N=47
CD8+CD279+, MFI	r=0.1436, p=0.3148, N=51	r=0.04207, p=0.7582, N=56	r=0.08438, p=0.5772, N=46	r=0.08986, p=0.5481, N=47
CD8+TIM3+, %	r=0.1868, p=0.1894, N=51	r=0.3359, p=0.0114, N=56	r=0.007279, p=0.9617, N=46	r=-0.008356, p=0.9555, N=47
CD8+TIM3+, MFI	r=0.2808, p=0.0459, N=51	r=0.4274, p=0.001, N=56	r=0.07633, p=0.6141, N=46	r=0.05207, p=0.7281, N=47
CD8+CTLA4+, %	r=0.05144, p=0.72, N=51	r=0.234, p=0.0826, N=56	r=0.08128, p=0.5913, N=46	r=0.08748, p=0.5587, N=47
CD8+CTLA4+, MFI	r=0.1934, p=0.2514, N=37	r=0.1993, p=0.2117, N=41	r=-0.1126, p=0.5194, N=35	r=-0.1208, p=0.4895, N=35
CD8+LAG3+, %	r=0.2146, p=0.1304, N=51	r=0.3022, p=0.0236, N=56	r=0.1251, p=0.4073, N=46	r=0.06906, p=0.6446, N=47

CD8+LAG3+, MFI	r=0.3763, p=0.0182, N=39	r=0.3014, p=0.0524, N=42	r=0.2732, p=0.1018, N=37	r=0.2955, p=0.0758, N=37
CD8+CD127+, %	r=-0.1466, p=0.3048, N=51	r=-0.07071, p=0.6045, N=56	r=-0.1306, p=0.3871, N=46	r=-0.09876, p=0.509, N=47
CD8+CD127+, MFI	r=-0.2729, p=0.0803, N=42	r=-0.1257, p=0.4052, N=46	r=-0.4244, p=0.0063, N=40	r=-0.3797, p=0.0157, N=40
CD8+TIGIT+, %	r=-0.06855, p=0.6327, N=51	r=0.1028, p=0.4507, N=56	r=-0.09432, p=0.5329, N=46	r=-0.01145, p=0.9391, N=47
CD8+TIGIT+, MFI	r=-0.005844, p=0.9711, N=41	r=0.2181, p=0.15, N=45	r=0.105, p=0.5247, N=39	r=0.1671, p=0.3092, N=39
CD8+CD160+, %	r=-0.02824, p=0.8441, N=51	r=0.09472, p=0.4875, N=56	r=-0.03636, p=0.8104, N=46	r=-0.03224, p=0.8297, N=47
CD8+CD160+, MFI	r=-0.09766, p=0.509, N=48	r=-0.05401, p=0.7038, N=52	r=-0.1246, p=0.4205, N=44	r=-0.1673, p=0.2778, N=44
CD8+CD244+, %	r=-0.1854, p=0.1928, N=51	r=-0.01942, p=0.887, N=56	r=-0.07449, p=0.6227, N=46	r=-0.08273, p=0.5804, N=47
CD8+CD244+, MFI	r=0.0854, p=0.5638, N=48	r=-0.1014, p=0.4702, N=53	r=0.08628, p=0.5776, N=44	r=0.005965, p=0.969, N=45
CD8+HLA-DR+, %	r=0.01632, p=0.9215, N=39	r=0.1793, p=0.2498, N=43	r=0.119, p=0.483, N=37	r=0.1331, p=0.4321, N=37
CD8+HLA-DR+, MFI	r=0.3127, p=0.0526, N=39	r=0.3766, p=0.0128, N=43	r=0.3149, p=0.0576, N=37	r=0.203, p=0.2283, N=37
CD8+CD38+, %	r=0.2218, p=0.1748, N=39	r=0.291, p=0.0583, N=43	r=0.07898, p=0.6422, N=37	r=0.01541, p=0.9279, N=37
CD8+CD38+, MFI	r=0.09666, p=0.5637, N=38	r=0.1238, p=0.4346, N=42	r=0.1286, p=0.4547, N=36	r=0.03373, p=0.8452, N=36
CD3+CD160+, %	r=-0.2344, p=0.0977, N=51	r=-0.1382, p=0.3099, N=56	r=-0.09457, p=0.5319, N=46	r=-0.07538, p=0.6146, N=47
CD3+CD160+, MFI	r=-0.1384, p=0.3378, N=50	r=-0.08164, p=0.5535, N=55	r=-0.145, p=0.3363, N=46	r=-0.1904, p=0.2, N=47
CD3+CD244+, %	r=-0.2879, p=0.0405, N=51	r=-0.02412, p=0.8599, N=56	r=-0.1439, p=0.3401, N=46	r=-0.1238, p=0.407, N=47
CD3+CD244+, MFI	r=0.08647, p=0.5463, N=51	r=-0.0994, p=0.4661, N=56	r=-0.0186, p=0.9023, N=46	r=-0.04173, p=0.7806, N=47
CD3+CD279+, %	r=0.1396, p=0.3337, N=50	r=0.1678, p=0.2207, N=55	r=0.1962, p=0.1966, N=45	r=0.1856, p=0.2168, N=46
CD3+CD279+, MFI	r=0.1609, p=0.2644, N=50	r=0.1485, p=0.2791, N=55	r=0.1026, p=0.5026, N=45	r=0.09734, p=0.5199, N=46
CD3+TIGIT+, %	r=-0.08385, p=0.5627, N=50	r=0.1247, p=0.3643, N=55	r=-0.05446, p=0.7224, N=45	r=-0.002344, p=0.9877, N=46
CD3+TIGIT+, MFI	r=0.01305, p=0.9291, N=49	r=0.08388, p=0.5465, N=54	r=0.1214, p=0.4326, N=44	r=0.1303, p=0.3937, N=45

Supplementary Table S3: The correlation between RA Disease Activity and Two Co-IRs expression on T cells

T cells Co-IRs	ESR	HS-CRP	Das28 ESR	Das28 CRP
CD4+CD279+TIM3+, %	r=0.2568, p=0.0689, N=51	r=0.4046, p=0.002, N=56	r=0.3842, p=0.0084, N=46	r=0.4391, p=0.002, N=47
CD4+CD279+TIM3+, CD279 MFI	r=0.088, p=0.552, N=48	r=0.0133, p=0.9247, N=53	r=-0.1323, p=0.392, N=44	r=-0.1258, p=0.4104, N=45
CD4+CD279+TIM3+, TIM3 MFI	r=0.1026, p=0.4877, N=48	r=0.006612, p=0.9625, N=53	r=-0.1341, p=0.3854, N=44	r=-0.2059, p=0.1747, N=45
CD4+CTLA4+LAG3+, %	r=-0.04291, p=0.765, N=51	r=0.1787, p=0.1877, N=56	r=-0.08826, p=0.5597, N=46	r=-0.05813, p=0.6979, N=47
CD4+CTLA4+LAG3+, CTLA4 MFI	r=0.1705, p=0.4152, N=25	r=0.1648, p=0.4021, N=28	r=-0.251, p=0.2367, N=24	r=-0.3496, p=0.094, N=24
CD4+CTLA4+LAG3+, LAG3 MFI	r=-0.1662, p=0.4271, N=25	r=-0.1943, p=0.3218, N=28	r=-0.07003, p=0.7451, N=24	r=-0.1092, p=0.6116, N=24
CD4+CD127+TIGIT+, %	r=0.01123, p=0.9376, N=51	r=-0.01918, p=0.8884, N=56	r=0.04226, p=0.7804, N=46	r=-0.02345, p=0.8757, N=47
CD4+CD127+TIGIT+, CD127 MFI	r=-0.2916, p=0.0644, N=41	r=-0.01087, p=0.9435, N=45	r=-0.1647, p=0.3163, N=39	r=-0.1135, p=0.4913, N=39
CD4+CD127+TIGIT+, TIGIT MFI	r=-0.01945, p=0.9039, N=41	r=0.269, p=0.074, N=45	r=0.05943, p=0.7193, N=39	r=0.002329, p=0.9888, N=39
CD8+CD279+TIM3+, %	r=0.06757, p=0.6376, N=51	r=0.03892, p=0.7758, N=56	r=0.2149, p=0.1516, N=46	r=0.1738, p=0.2428, N=47
CD8+CD279+TIM3+, CD279 MFI	r=0.02654, p=0.8779, N=36	r=0.1183, p=0.4614, N=41	r=0.002904, p=0.987, N=34	r=-0.02787, p=0.8737, N=35
CD8+CD279+TIM3+, TIM3 MFI	r=-0.1042, p=0.5452, N=36	r=0.06655, p=0.6793, N=41	r=-0.01666, p=0.9255, N=34	r=-0.08306, p=0.6352, N=35
CD8+CTLA4+LAG3+, %	r=-0.1679, p=0.239, N=51	r=-0.01955, p=0.8863, N=56	r=-0.229, p=0.1258, N=46	r=-0.1983, p=0.1816, N=47
CD8+CTLA4+LAG3+, CTLA4 MFI	r=-0.04184, p=0.9219, N=9	r=-0.01667, p=0.9816, N=9	r=-0.05988, p=0.8987, N=8	r=0.1667, p=0.7033, N=8
CD8+CTLA4+LAG3+, LAG3 MFI	r=-0.3515, p=0.3502, N=9	r=-0.2, p=0.6134, N=9	r=-0.3353, p=0.4129, N=8	r=-0.4762, p=0.2431, N=8
CD8+CD127+TIGIT+, %	r=-0.2952, p=0.0355, N=51	r=-0.1134, p=0.4052, N=56	r=-0.2292, p=0.1255, N=46	r=-0.1332, p=0.3721, N=47
CD8+CD127+TIGIT+, CD127 MFI	r=-0.2207, p=0.1656, N=41	r=-0.05573, p=0.7161, N=45	r=-0.2367, p=0.1469, N=39	r=-0.2144, p=0.19, N=39
CD8+CD127+TIGIT+, TIGIT MFI	r=-0.1348, p=0.4009, N=41	r=0.1845, p=0.2251, N=45	r=-0.06115, p=0.7115, N=39	r=-0.0656, p=0.6915, N=39
CD8+CD160+CD244+, %	r=-0.02507, p=0.8628, N=50	r=-0.01259, p=0.9273, N=55	r=0.05661, p=0.7119, N=45	r=0.03581, p=0.8132, N=46
CD8+CD160+CD244+, CD160 MFI	r=0.03832, p=0.8072, N=43	r=0.07805, p=0.602, N=47	r=-0.05526, p=0.7348, N=40	r=-0.1112, p=0.4946, N=40

CD8+CD160+CD244+, CD244 MFI	r=0.05654, p=0.7188, N=43	r=-0.0314, p=0.8341, N=47	r=-0.07323, p=0.6534, N=40	r=-0.09861, p=0.5449, N=40
CD8+HLA-DR+CD127+, %	r=-0.2214, p=0.3622, N=19	r=-0.1925, p=0.4033, N=21	r=-0.106, p=0.6944, N=16	r=0.0515, p=0.8442, N=17
CD8+HLA-DR+CD127+, HLA-DR MFI	r=-0.1084, p=0.6685, N=18	r=0.0406, p=0.865, N=20	r=-0.02857, p=0.9234, N=15	r=0.1029, p=0.7049, N=16
CD8+HLA-DR+CD127+, CD127 MFI	r=-0.1518, p=0.5477, N=18	r=-0.09023, p=0.7052, N=20	r=0, p>0.9999, N=15	r=-0.07647, p=0.7798, N=16
CD8+HLA-DR+CD38+, %	r=0.226, p=0.1145, N=50	r=0.3034, p=0.0243, N=55	r=0.1956, p=0.1978, N=45	r=0.2224, p=0.1374, N=46
CD8+HLA-DR+CD38+, HLA-DR MFI	r=-0.001202, p=0.9934, N=50	r=0.01468, p=0.9153, N=55	r=0.1709, p=0.2618, N=45	r=0.09487, p=0.5306, N=46
CD8+HLA-DR+CD38+, CD38 MFI	r=-0.09162, p=0.5269, N=50	r=0.003031, p=0.9825, N=55	r=0.0147, p=0.9237, N=45	r=-0.04932, p=0.7448, N=46
CD3+CD160+CD244+, %	r=-0.317, p=0.0234, N=51	r=-0.144, p=0.2897, N=56	r=-0.1993, p=0.1842, N=46	r=-0.1455, p=0.3293, N=47
CD3+CD160+CD244+, CD160 MFI	r=-0.215, p=0.1466, N=47	r=-0.2547, p=0.0685, N=52	r=-0.2323, p=0.1292, N=44	r=-0.2454, p=0.1042, N=45
CD3+CD160+CD244+, CD244 MFI	r=-0.1121, p=0.4529, N=47	r=-0.1219, p=0.3894, N=52	r=-0.1792, p=0.2444, N=44	r=-0.1835, p=0.2276, N=45
CD3+CD279+TIGIT+, %	r=0.2149, p=0.134, N=50	r=0.2045, p=0.1341, N=55	r=0.2096, p=0.1671, N=45	r=0.205, p=0.1716, N=46
CD3+CD279+TIGIT+, CD279 MFI	r=-0.1271, p=0.3792, N=50	r=-0.1313, p=0.3394, N=55	r=-0.1019, p=0.5054, N=45	r=-0.05665, p=0.7084, N=46
CD3+CD279+TIGIT+, TIGIT MFI	r=-0.1995, p=0.1648, N=50	r=-0.02864, p=0.8355, N=55	r=-0.0537, p=0.7261, N=45	r=-0.01671, p=0.9122, N=46
